# O022. Migraineurs: seriously ill or basically healthy?

**DOI:** 10.1186/1129-2377-16-S1-A66

**Published:** 2015-09-28

**Authors:** Alessandro Panconesi, Maria L Bartolozzi, Leonello Guidi

**Affiliations:** Headache Center, Department of Neurology, Health Authority 11, Empoli, Italy

## Background

Some epidemiological studies report many comorbidities in migraine, mostly identified through questionnaires and based on self-reported diagnosis[1]. Pharmacoepidemiological studies have also identified some comorbidities. We evaluated the migraine comorbidities using and comparing data derived by different methods of study, with the aim of minimizing the variability of results.

## Materials and methods

We evaluated prospectively the prevalence of some comorbidities in the first 1,000 migraine patients (aged 15-65 years), classified according to the IHS diagnostic criteria, presenting to the Headache Center (HC) in the period 2011 to 2013 and resident in the district of the Empoli Health Authority (HA11). In addition to a detailed semistructured face to face interview, health information was researched in the database of specialist visits, hospitalization or the Emergency Department. Drug prescription rates of the HA11 pharmaceutical database (PD) of 155,829 residents aged 15-65 years were also analyzed, as were the subgroup of 1,108 triptan users.

To increase the strength of the study, the data was compared to a large database of Health Search (HS) of the Research Institute of the Italian Society of General Practice, to which general practitioners contributed with shared modalities for the regular and complete recording of the principal health data[2].

## Results

In our clinical survey (780 females, 220 males; mean age 39.5 years; 866 episodic migraine, 134 chronic migraine) the prevalence of treated diseases were: depression 5.6%, hypertension, 9.3%, diabetes 1.2%, asthma 3.3%, hypotiroidism 6.4%, hypertiroidism 0.3%, hypercholesterolemia 0.5%. These percentages are not superior to drug prescription rate found in the PD in the general population. A higher prescription rate for some chronic diseases was found in triptan users in comparison with the general population and migraine patients (HC), but this may be due to drugs also used in migraine prevention.

The HS database, covering 893,870 subjects aged 14-85 years, showed an increased percentage of depression (20.0 vs 5.5) and hypotiroidism (6.1 vs 3.7) in migraine patients, but not of hypertension or diabetes[2].

## Conclusions

This was the first study on migraine comorbidities utilizing three different accurate databases (HC, PD, HS), within the same country. Our study does not confirm that many of the comorbidities reported are associated with migraine, in particular those involved in cardiovascular risk. The contrasting evidence in the literature could be due to the bias intrinsic in the method of study: group, age not comparable, sex differences, marker drugs used off label, and uncertainness of diagnosis.

Written informed consent to publication was obtained from the patient(s).
